# Supplementary material for: Cryo-EM structure of cyanophage P-SCSP1u offers insights into DNA gating and evolution of T7-like viruses
Source: Nat Commun. 2023 Oct 13;14:6438. doi: 10.1038/s41467-023-42258-7 (PMC10575957; doi:10.1038/s41467-023-42258-7)
Supplement: Supplementary file 1 — Supplementary Information [file 41467_2023_42258_MOESM1_ESM.pdf]

Supplementary Information for

Cryo-EM structure of cyanophage P-SCSP1u offers insights into DNA gating and evolution of T7-like viruses

Lanlan Cai, Hang Liu, Wen Zhang, Shiwei Xiao, Qinglu Zeng, Shangyu Dang

To whom correspondence may be addressed. Email: zeng@ust.hk (Q.Z.); sdang@ust.hk (S.D.)

This file includes:

- Supplementary Figures 1 to 6
- Supplementary Table 1

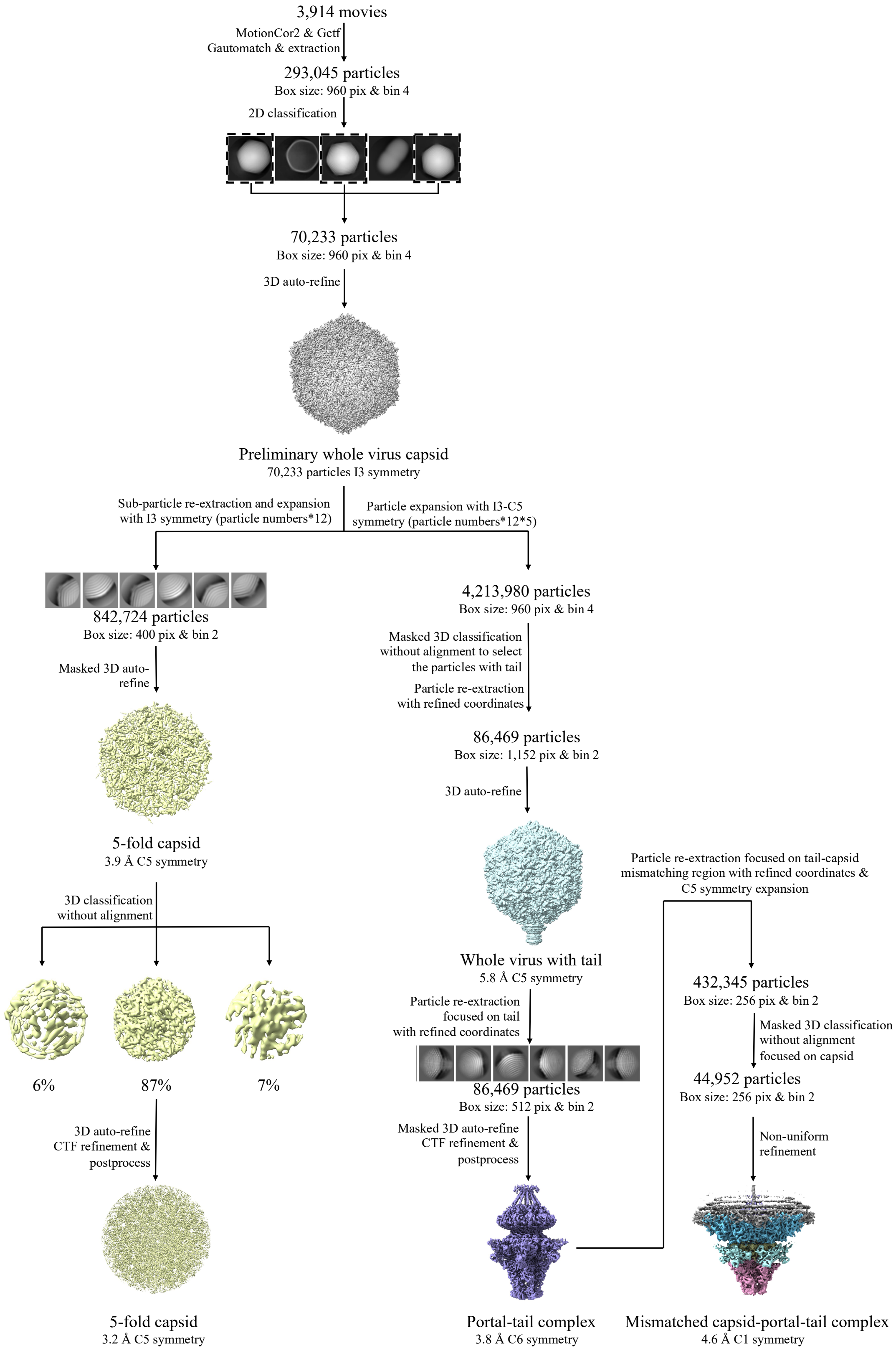

Supplementary Figure 1. Cryo-EM data processing of the P-SCSP1u virion. The capsid and portal-tail complex were reconstructed using different strategies. The resolutions and the number of phage particles used are indicated.

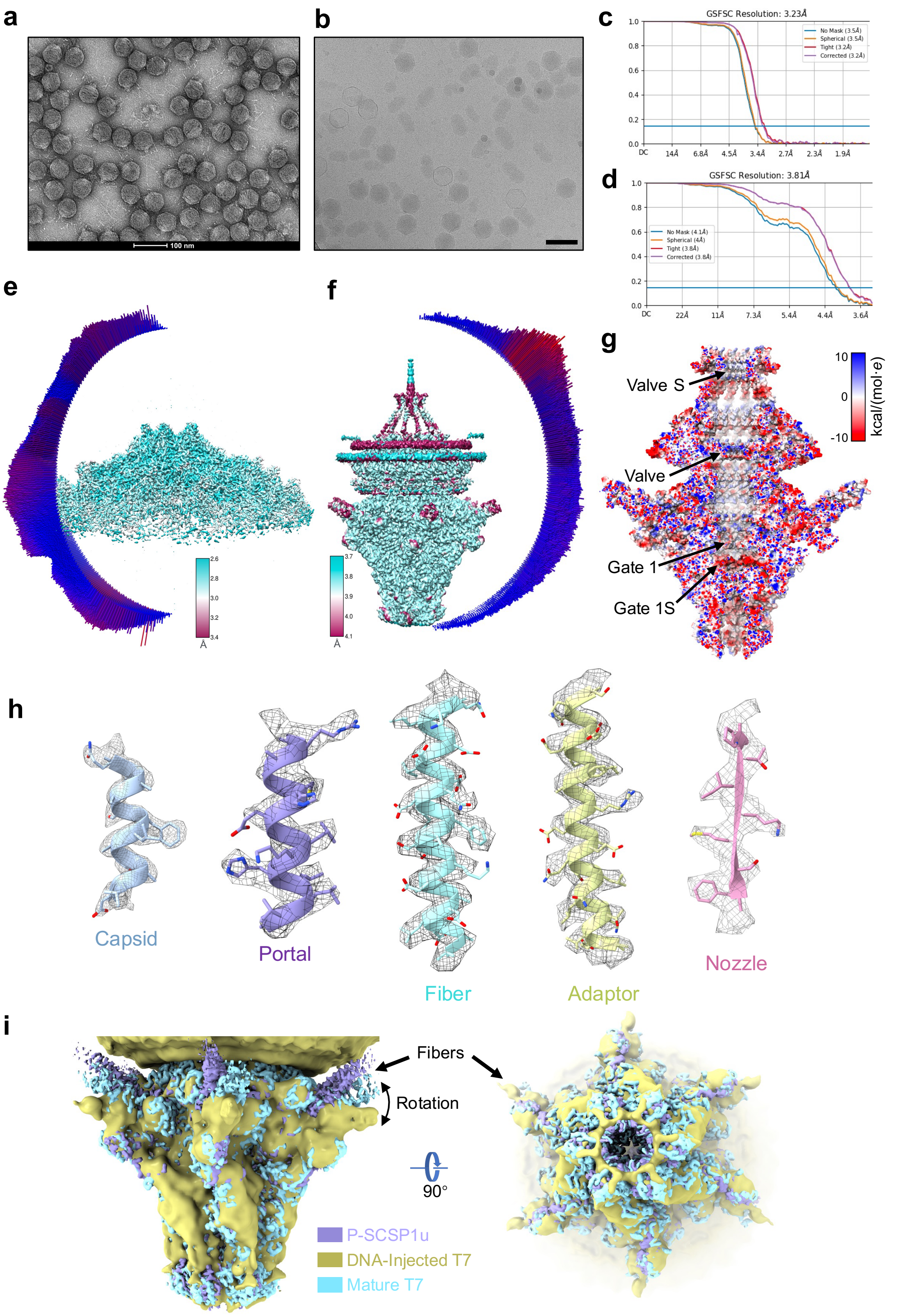

Supplementary Figure 2. Cryo-EM analysis of the P-SCSP1u virion. a, A representative negative stain micrograph of P-SCSP1 virions. Scale bar, 100 nm. **b,** A representative cryo-EM micrograph of P-SCSP1 virions. Scale bar, 100 nm. **c-d,** FSC curves of the 3D reconstruction of the P-SCSP1u major capsid protein (**c**) and portal-tail complex (**d**). The blue line corresponds to FSC = 0.143. **e-f,** The local resoulution

map and angular distribution of the final 3D reconstruction of the P-SCSP1u major capsid protein (**e**) and portal-tail complex (**f**). **g**, The electrostatic potential surface of the portal-tail complex. Newly identified valve (Valve S) and gate (Gate 1S) in this study, together with previously identified Valve and Gate 1, are indicated. **h**, The cryo-EM densities of representative fragments of the capsid, portal, fiber, adaptor, and nozzle of P-SCSP1u. The density is shown as grey mesh, and the corresponding structural model is colored differently. **i**, Comparison of the portal-tail complex between P-SCSP1u and T7. The cryo-EM density map of the P-SCSP1u (purple) was overlaid with those of DNA-injected T7 (yellow, EMDB-31322) and mature T7 (cyan, EMDB-31319) to show the angle differences of fibers at different states.

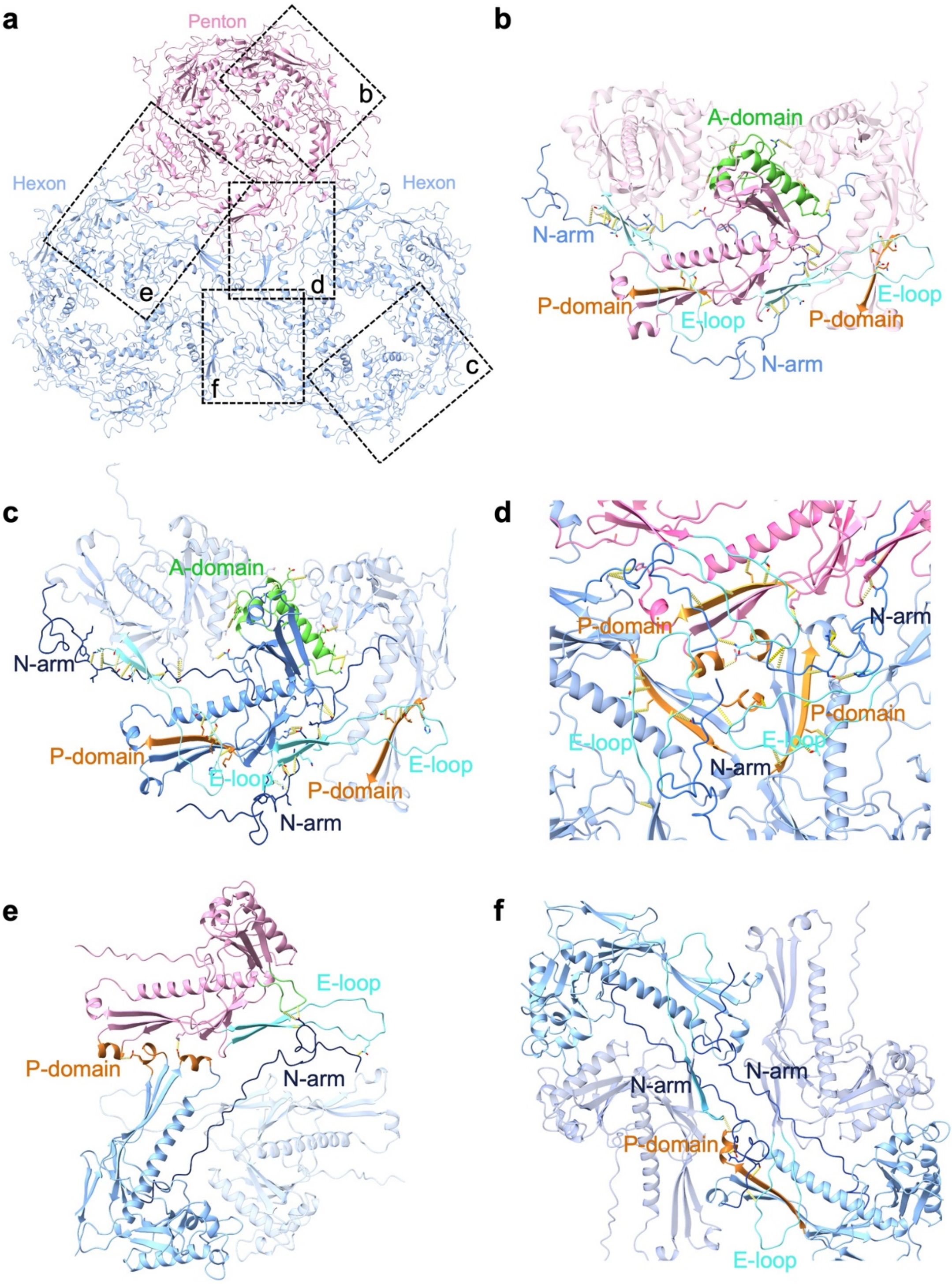

Supplementary Figure 3. The subunit interactions within/between capsomeres of the P-SCSP1u capsid. a, The overall view of one penton (magenta) and two hexons (blue) of the P-SCSP1u capsid are shown. The interaction details highlighted with dashed boxes are shown in higher magnification in the following panels, including interactions of intra-penton (**b**), intra-hexon (**c**), penton-hexon (**d** and **e**), and inter-hexons (**f**). Domains involved in interactions were labeled and colored differently. The hydrogen bonds were indicated with yellow dashed lines.

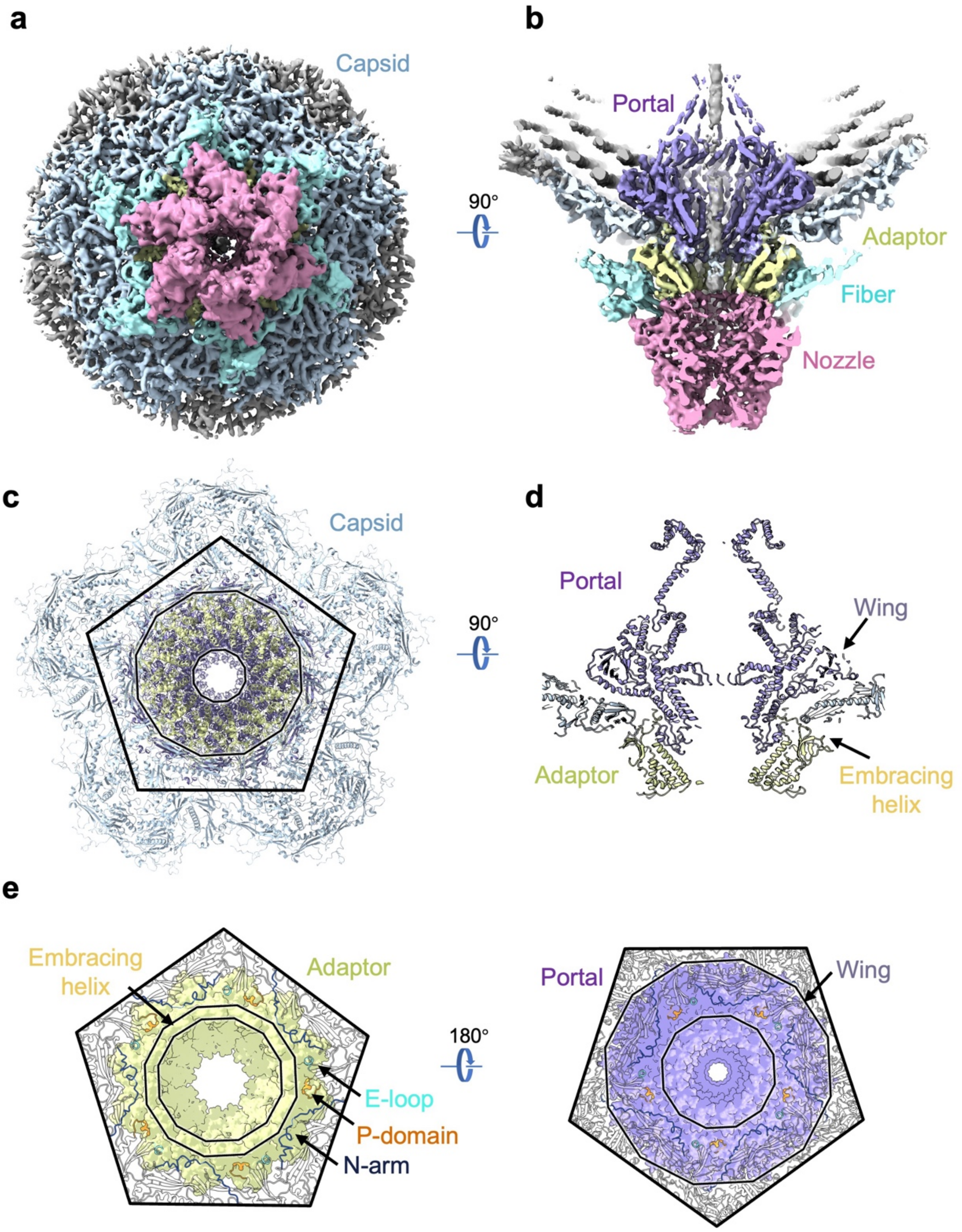

Supplementary Figure 4. The structural details of the mismatched capsid-portal-tail complex. The density map of the mismatched capsid-portal-tail complex was shown as the top view (**a**) and section view (**b**). The structural models of the mismatched capsid-portal-adaptor complex are shown as the top view (**c**) and section view (**d**). **e**, The key interactive regions between the capsid and the portal-tail complex. The maps and models were colored differently according to the protein components. The black pentagon and dodecagon indicated the C5 symmetry of the capsid and the C12 symmetry of the portal-adaptor complex.

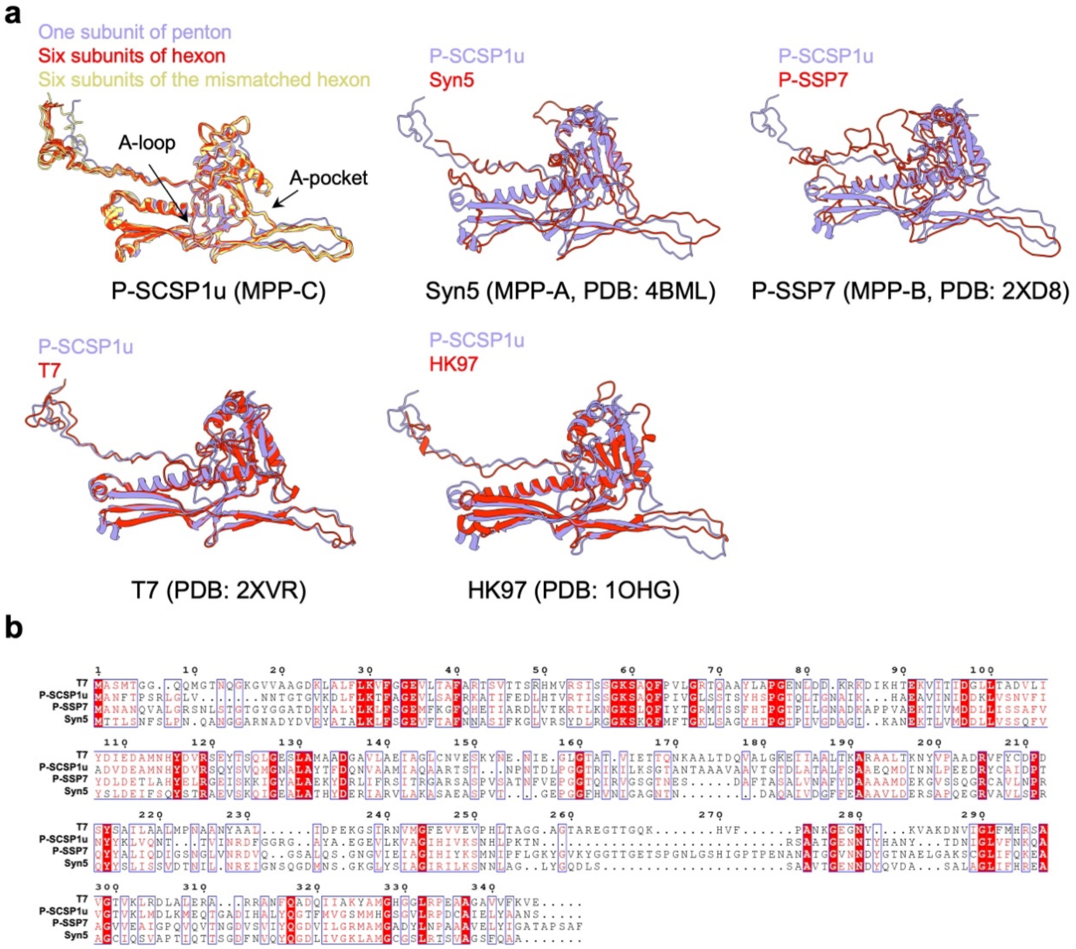

Supplementary Figure 5. Comparison of the major capsid proteins among different podoviruses. a, Structural comparison of the major capsid protein between penton and hexon of P-SCSP1u, P-SCSP1u and Syn5 (MPP-A phage, PDB: 4BML), P-SSP7 (MPP-B phage, PDB: 2XD8), T7 (PDB: 2XVR), as well as HK97 (PDB: 1OHG). The A-pocket and A-loop that are characteristics of T7-like phages are indicated. **b**, Sequence alignments of the capsid proteins among T7 and T7-like cyanophages.

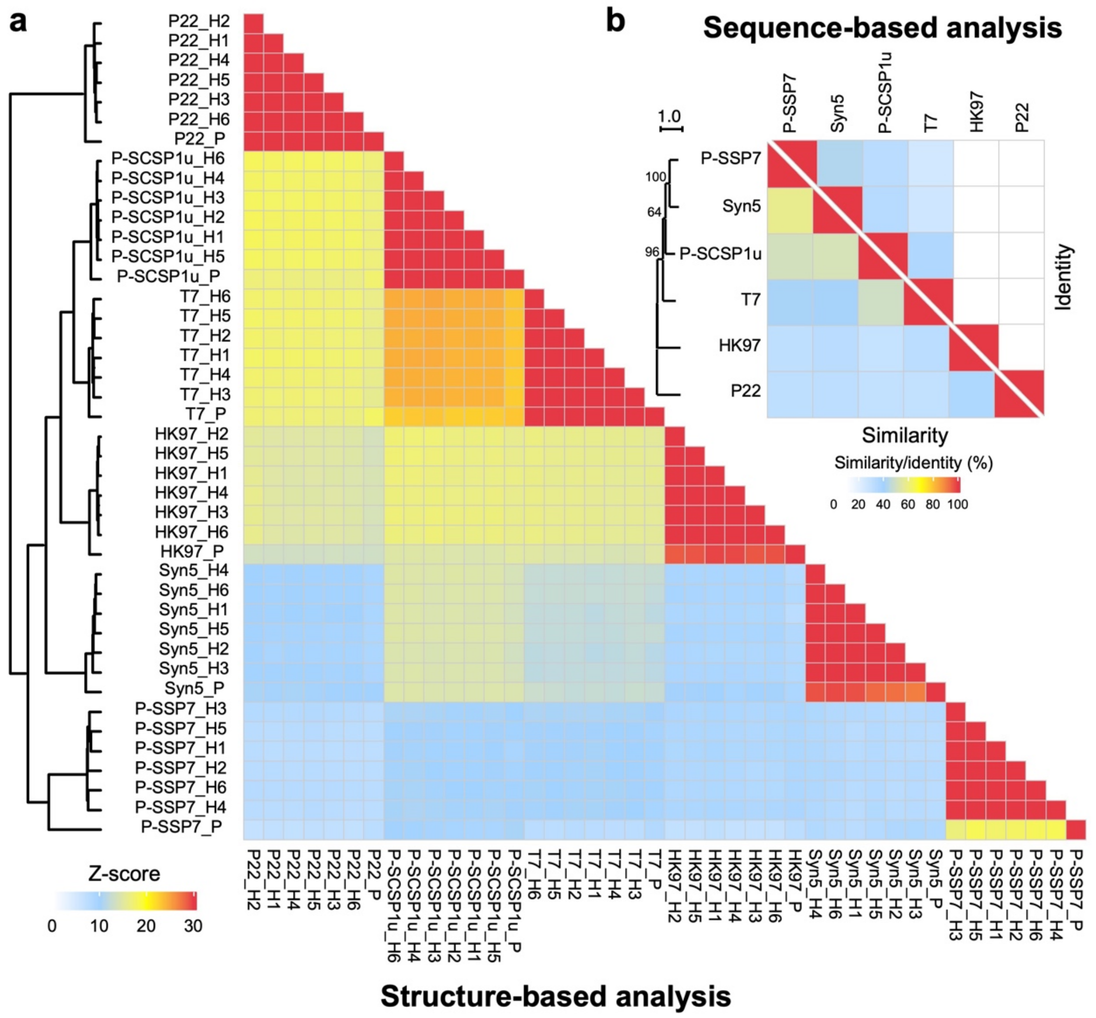

Supplementary Figure 6. Comparison of the capsid proteins among different podoviruses (including T7, T7-like cyanophages, HK97, and *Salmonella* phage P22) based on structure (a) and sequence (b) analyses. a, Hierarchical clustering and structural similarity matrix (DALI Z-scores) of the capsid protein subunits from different phages. Seven capsid protein subunits in the viral asymmetric unit of different phages are used for pairwise comparison. Six subunits of one hexon are denoted as H1 to H6, respectively, and one subunit of penton is denoted as P. Z-scores between different proteins were calculated using the DALI server. Higher scores indicate better structural similarity. The similarity matrix is used for hierarchical clustering analysis. **b**, The phylogenetic tree on the left is generated from the amino acid sequences of the capsid protein using MEGA software. Bootstrap values for 1,000 replicates are indicated by numbers at the nodes. The scale bar indicates amino acid substitution. The lower-left and upper-right of the heatmap indicate sequence similarities performed by SnapGene and sequence identities performed by BLASTp, respectively. Source data are provided as a Source Data file.

Supplementary Table 1. Cryo-EM data collection, refinement and validation statistics.

	P-SCSP1u capsid (gp19) (EMDB-35174) (PDB 8I4L)	P-SCSP1u portal (gp16) (EMDB-35175) (PDB 8I4M)	P-SCSP1u adaptor (gp22) (EMDB-35175) (PDB 8I4M)	P-SCSP1u nozzle (gp23) (EMDB-35175) (PDB 8I4M)	P-SCSP1u fiber (gp28) (EMDB-35175) (PDB 8I4M)
**Data collection and processing**					
Magnification	50,000		50,000		
Voltage (kV)	300		300		
Electron exposure (e–/Å^2)	50		50		
Defocus range (μm)	-1.0~-2.5		-1.0~-2.5		
Pixel size (Å)	0.85		0.85		
Symmetry imposed	C5		C6		
Initial particle images (no.)	293,045		293,045		
Final particle images (no.)	732,864*		86,469*		
Map resolution (Å)	3.23		3.81		
FSC threshold	0.143		0.143		
Map resolution range (Å)	2.6-3.4		3.7-4.1		
**Refinement**					
Initial model used (PDB code)	SWISS-MODEL		AlphaFOLD		
Model resolution (Å)	3.35	5.06	4.06	3.93	4.28
FSC threshold	0.5	0.5	0.5	0.5	0.5
Map sharpening *B* factor (Å^2)	-178	-180	-180	-180	-180
Model composition					
Non-hydrogen atoms	17,213	52,512	19,984	36,954	26,064
Protein residues	2,289	6,780	2,376	4,836	3,456
Ligands	0	0	0	0	0
B factors (Å^2)					
Protein	58.95	124.04	88.43	94.62	115.54
Ligand	N/A	N/A	N/A	N/A	N/A
R.m.s. deviations					
Bond lengths (Å)	0.003	0.004	0.004	0.004	0.005
Bond angles (°)	0.575	0.974	0.887	0.973	1.05
Validation					
MolProbity score	1.61	2.68	1.82	1.86	2.26
Clashscore	4.89	64.19	14.61	10.81	19.69
Poor rotamers (%)	0.26	0.65	0.21	0.43	0.35
Ramachandran plot					
Favored (%)	94.86	94.03	97.19	95.52	92.49
Allowed (%)	5.14	5.79	2.81	4.48	7.51
Disallowed (%)	0	0.18	0	0	0

*Particles number after symmetry expansion.

Supplementary Table 2. Reference structures of capsid used for structural comparison and DALI analysis.

Protein	PDB
P22	5UU5
P-SCSP1u	8I4L
T7	2XVR
HK97	1OHG
Syn5	4BML
P-SSP7	2XDB
