# Supplementary material for: Cryo-EM structure of cyanophage P-SCSP1u offers insights into DNA gating and evolution of T7-like viruses
Source: Nat Commun. 2023 Oct 13;14:6438. doi: 10.1038/s41467-023-42258-7 (PMC10575957; doi:10.1038/s41467-023-42258-7)
Supplement: Supplementary file 3 — Reporting Summary [file 41467_2023_42258_MOESM3_ESM.pdf]

nature portfolio

Corresponding author(s): Shangyu Dang, Qinglu Zeng

Last updated by author(s): Sep 25, 2023

Reporting Summary

Nature Portfolio wishes to improve the reproducibility of the work that we publish. This form provides structure for consistency and transparency in reporting. For further information on Nature Portfolio policies, see our Editorial Policies and the Editorial Policy Checklist.

Statistics

For all statistical analyses, confirm that the following items are present in the figure legend, table legend, main text, or Methods section.

n/a	Confirmed	
☒	☐	The exact sample size (*n*) for each experimental group/condition, given as a discrete number and unit of measurement
☒	☐	A statement on whether measurements were taken from distinct samples or whether the same sample was measured repeatedly
☒	☐	The statistical test(s) used AND whether they are one- or two-sided<br>*Only common tests should be described solely by name; describe more complex techniques in the Methods section.*
☒	☐	A description of all covariates tested
☒	☐	A description of any assumptions or corrections, such as tests of normality and adjustment for multiple comparisons
☒	☐	A full description of the statistical parameters including central tendency (e.g. means) or other basic estimates (e.g. regression coefficient) AND variation (e.g. standard deviation) or associated estimates of uncertainty (e.g. confidence intervals)
☒	☐	For null hypothesis testing, the test statistic (e.g. *F*, *t*, *r*) with confidence intervals, effect sizes, degrees of freedom and *P* value noted<br>*Give P values as exact values whenever suitable.*
☒	☐	For Bayesian analysis, information on the choice of priors and Markov chain Monte Carlo settings
☒	☐	For hierarchical and complex designs, identification of the appropriate level for tests and full reporting of outcomes
☒	☐	Estimates of effect sizes (e.g. Cohen's *d*, Pearson's *r*), indicating how they were calculated

Our web collection on statistics for biologists contains articles on many of the points above.

Software and code

Policy information about availability of computer code

Data collection	EPU V2.10.0
Data analysis	MotionCor2 v1.4.0, GCTF, REION 3.1, CryoSPARC v2.15.0, UCSF Chimera v1.11.2, Chimera X v1.3, COOT v0.9.5, Phenix v1.20, DALI

For manuscripts utilizing custom algorithms or software that are central to the research but not yet described in published literature, software must be made available to editors and reviewers. We strongly encourage code deposition in a community repository (e.g. GitHub). See the Nature Portfolio guidelines for submitting code & software for further information.

Data

Policy information about availability of data

All manuscripts must include a data availability statement. This statement should provide the following information, where applicable:

- Accession codes, unique identifiers, or web links for publicly available datasets
- A description of any restrictions on data availability
- For clinical datasets or third party data, please ensure that the statement adheres to our policy

Cryo-EM density maps of P-SCSP1u cyanophage have been deposited in the Electron Microscopy Data Bank under accession code EMD-35174 [https://www.ebi.ac.uk/emdb/EMD-35174] (capsid), EMD-35175 [https://www.ebi.ac.uk/emdb/EMD-35175] (portal-tail complex). Atomic coordinates have been deposited in the Protein Data Bank under accession code 8I4L [http://doi.org/10.2210/pdb8I4L/pdb] (capsid), 8I4M [http://doi.org/10.2210/pdb8I4M/pdb] (portal-tail complex). The DALI Z-score (Supplementary Figure 6a) and identify and similarity of sequence alignment (Supplementary Figure 6b) have been provided in the source data.

Research involving human participants, their data, or biological material

Policy information about studies with human participants or human data. See also policy information about sex, gender (identity/presentation), and sexual orientation and race, ethnicity and racism.

Reporting on sex and gender	Not applied to this study, which focuses on structural determination and analysis of the cyanophage P-SCSP1u.
Reporting on race, ethnicity, or other socially relevant groupings	Not applied to this study, which focuses on structural determination and analysis of the cyanophage P-SCSP1u.
Population characteristics	Not applied to this study, which focuses on structural determination and analysis of the cyanophage P-SCSP1u.
Recruitment	Not applied to this study, which focuses on structural determination and analysis of the cyanophage P-SCSP1u.
Ethics oversight	Not applied to this study, which focuses on structural determination and analysis of the cyanophage P-SCSP1u.

Note that full information on the approval of the study protocol must also be provided in the manuscript.

Field-specific reporting

Please select the one below that is the best fit for your research. If you are not sure, read the appropriate sections before making your selection.

[x] Life sciences [ ] Behavioural & social sciences [ ] Ecological, evolutionary & environmental sciences

For a reference copy of the document with all sections, see nature.com/documents/nr-reporting-summary-flat.pdf

Life sciences study design

All studies must disclose on these points even when the disclosure is negative.

Sample size	No statistical methods were used to predetermine sample size in this study. For cryo-EM study, the number of collected micrograph were determined based on the target resolution and availability of holes in the grids.
Data exclusions	Raw micrographs were screened manually to exclude bad ones based on the quality of the images. Particles grouped into bad classes after 2D and 3D classification were excluded from the final map calculation.
Replication	Single-particle cryo-EM analysis relies on averaging over a large number of good particles sorted out through data processing.
Randomization	Particles were randomly split into 2 groups following the gold standard FSC for resolution estimation.
Blinding	Investigators were not blinded during cryo-EM data collection and processing, because visual inspection is necessary for structural determination and analysis in this study.

Reporting for specific materials, systems and methods

We require information from authors about some types of materials, experimental systems and methods used in many studies. Here, indicate whether each material, system or method listed is relevant to your study. If you are not sure if a list item applies to your research, read the appropriate section before selecting a response.

Materials & experimental systems

n/a	Involved in the study
x	Antibodies
x	Eukaryotic cell lines
x	Palaeontology and archaeology
x	Animals and other organisms
x	Clinical data
x	Dual use research of concern
x	Plants

Methods

n/a	Involved in the study
x	ChIP-seq
x	Flow cytometry
x	MRI-based neuroimaging
